# A Bayesian mathematical model of motor and cognitive outcomes in Parkinson’s disease

**DOI:** 10.1371/journal.pone.0178982

**Published:** 2017-06-12

**Authors:** Boris Hayete, Diane Wuest, Jason Laramie, Paul McDonagh, Bruce Church, Shirley Eberly, Anthony Lang, Kenneth Marek, Karl Runge, Ira Shoulson, Andrew Singleton, Caroline Tanner, Iya Khalil, Ajay Verma, Bernard Ravina

**Affiliations:** 1 GNS Healthcare, Cambridge, Massachusetts, United States of America; 2 Novartis, Cambridge, Massachusetts, United States of America; 3 Alexion Pharmaceuticals, Cambridge, Massachusetts, United States of America; 4 University of Rochester, Rochester, New York, United States of America; 5 Morton and Gloria Movement Disorders Clinic and the Edmond J. Safra Program in Parkinson’s Disease, Toronto Western Hospital and the University of Toronto, Toronto, Ontario, Canada; 6 Institute for Neurodegenerative Disorders, New Haven, Connecticut, United States of America; 7 Georgetown University, Washington, DC, United States of America; 8 National Institute on Aging, NIH, Bethesda, Maryland, United States of America; 9 University of San Francisco & San Francisco Veterans Affairs Medical Center, San Francisco, California, United States of America; 10 Biogen Idec, Cambridge, Massachusetts, United States of America; 11 Voyager Therapeutics, Cambridge, Massachusetts, United States of America; Oslo Universitetssykehus, NORWAY

## Abstract

**Background:**

There are few established predictors of the clinical course of PD. Prognostic markers would be useful for clinical care and research.

**Objective:**

To identify predictors of long-term motor and cognitive outcomes and rate of progression in PD.

**Methods:**

Newly diagnosed PD participants were followed for 7 years in a prospective study, conducted at 55 centers in the United States and Canada. Analyses were conducted in 244 participants with complete demographic, clinical, genetic, and dopamine transporter imaging data. Machine learning dynamic Bayesian graphical models were used to identify and simulate predictors and outcomes. The outcomes rate of cognition changes are assessed by the Montreal Cognitive Assessment scores, and rate of motor changes are assessed by UPDRS part-III.

**Results:**

The most robust and consistent longitudinal predictors of cognitive function included older age, baseline Unified Parkinson’s Disease Rating Scale (UPDRS) parts I and II, Schwab and England activities of daily living scale, striatal dopamine transporter binding, and SNP rs11724635 in the gene BST1. The most consistent predictor of UPDRS part III was baseline level of activities of daily living (part II). Key findings were replicated using long-term data from an independent cohort study.

**Conclusions:**

Baseline function near the time of Parkinson’s disease diagnosis, as measured by activities of daily living, is a consistent predictor of long-term motor and cognitive outcomes. Additional predictors identified may further characterize the expected course of Parkinson’s disease and suggest mechanisms underlying disease progression. The prognostic model developed in this study can be used to simulate the effects of the prognostic variables on motor and cognitive outcomes, and can be replicated and refined with data from independent longitudinal studies.

## Introduction

Parkinson’s disease (PD) is characterized by progressive motor and non-motor manifestations including cognitive and behavioral impairment. The course of disease is highly variable and there are no established prognostic biomarkers or predictive models. We sought to develop a predictive model of motor and cognitive outcomes using a unique data set from the Longitudinal and Biomarker Study in PD (LABS-PD)[1]. This study followed de novo PD participants for up to 7 years and combined comprehensive clinical assessments, genetic risk markers, and dopamine transporter (DAT) imaging data.

Prior studies of PD progression focused on hypothesis driven analyses to show associations of select demographic, clinical, or imaging features with clinical outcomes. While this approach is useful, the large amount of different data types and potential permutations of variables in LABS-PD and other ongoing cohort studies make it challenging to develop predictive models based only on hypotheses. Much of the data may go unused, and the selection of variables to be analyzed may reflect the biases of the investigators or findings in the literature.

Some of these challenges can be overcome by using machine learning methods to build models of progression and clinical outcomes. Machine learning tools can empirically detect key features among complex datasets. We used a supercomputer-enabled machine learning platform, Reverse Engineering and Forward Simulation (REFS), to construct a predictive dynamic Bayesian graphical model of clinical outcomes and progression based on the LABS-PD data. This approach allowed empirical integration of different variables and their combination into the most parsimonious set of explanations and has been previously used for a rheumatoid arthritis disease model[2] as well as in modeling cell cycle progression and survival in breast cancer[3].

## Methods

Participants were drawn from the LABS-PD study. The development of this cohort and the schedule of assessments have been previously described[1]. Briefly, newly diagnosed, untreated PD subjects who had originally participated in a clinical trial, which was concluded prematurely due to futility in achieving efficacy, consented to be enrolled in an observational study of PD and followed with annual assessments. Three hundred and eighty participants who donated blood samples for DNA and had DAT imaging consistent with PD, were included in this modeling data set. Institutional Review Board approval was obtained for each participating site.

University of Rochester Institutional Review Board approval was obtained for each participating site. Further more, UofR IRB approved this specific study. No such approval was needed for the publicly available PPMI data used for validation. Both studies collected participants’ written consent at the time of enrollment. No personally identifiable information, such as birth date (beyond year of birth), was used in this study from either the PPMI or the LABS-PD cohort. None of the authors were directly involved in the patients’ medical treatment. No site investigators participated in this study.

### Model variables

The main model outcomes of interest were motor function and cognitive function at years 5–7 of follow-up. The primary outcome was the Unified Parkinson’s Disease Rating Scale (UPDRS) parts I-IV[4] with a focus on part III (motor). The primary cognitive outcome was the Montreal Cognitive Assessment[5]. Outcomes were measured annually by a trained investigator at the participating Parkinson Study Group site. The MoCA was not assessed at baseline, but was added to the assessment battery at year 4. Levodopa equivalents[6] at the time of outcome assessment (years 5–7) were included in the model to account for dopaminergic treatment during the assessment period.

Predictive variables included demographics, disease duration, and baseline measures of motor and non-motor function in the de novo period before requiring dopaminergic therapy, baseline DAT brain imaging, and genetic markers. Motor assessments included the UPDRS parts I-III, Modified Hoehn and Yahr Stage (H&Y)[7], and the Schwab and England Activities of Daily Living Scale (S/E ADL)[8]. Non-motor clinical assessments included the Mini Mental Status Exam (MMSE)[9], Beck Depression Inventory[10], and Parkinson’s Disease Quality of Life (PDQ39) Scale[11].

[^123^I][β]-CIT and SPECT brain imaging scans were collected as previously described[12]. The [^123^I][β]-CIT tracer binds to the DAT on presynaptic dopaminergic nerve terminals in the caudate and putamen. The measures of interest were the striatal binding ratio (SBR) at baseline and the annual percent change in β-CIT SBR between baseline and 22-months. The primary quantitative imaging measure, the specific non-displaceable striatal uptake, was determined through a standardized analysis method using the occipital cortex as the the reference region[13,14]. Based on a separate database of 100 healthy participants, baseline scans were categorized as either DAT deficient (≤80% age-expected lowest putamen β-CIT uptake) or not DAT deficient (Scans Without Evidence of Dopaminergic Deficit, SWEDD, >80% age-expected lowest putamen β-CIT uptake)[15]. Only participants with DAT deficient scans consistent with PD were included in the analyses.

Genotyping was conducted with the Illumina BeadChip used in prior GWAS meta-analyses (Immunochip) to assess risk for PD. Methods have previously been reported for imputation of sequence variants[16]. Additional genotypes of interest not on the Immunochip were added to the analyses including, ApoE isoform, single nucleotide polymorphisms (SNPs) in the MAPT gene (rs199533 and rs175563986), alpha-synculein (rs2736990), and park 16 (rs823156). Only SNPs with a minor allele frequency of >5% were included in the modeling data frame.

### Data processing

**Fig 1:**
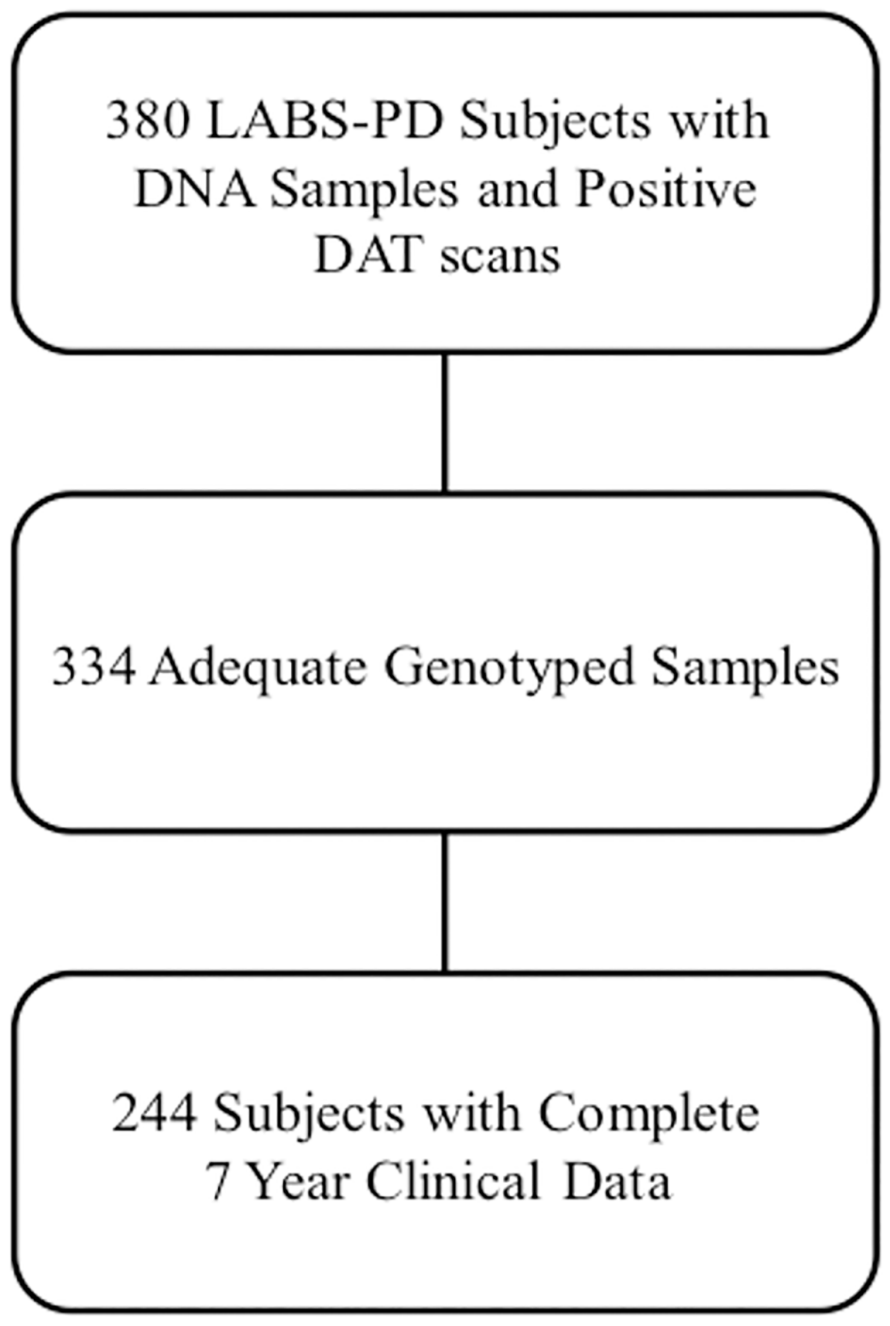
Participant selection for the study. Participants were included in the analysis if they had adequate genotype and 7 year clinical data.

For single nucleotide polymorphism (SNP) processing, the 334 available genotypes were matched to the 270 participants who had mostly complete datasets (less than 20% missing data), which reduced the number of participants available for modeling to 244 (Fig 1). The genotypes of the 244 participants were pruned according to the following criteria: (1) Minor allele frequency greater than 5%, (2) missing fraction less than 5%, and (3) Hardy-Weinberg equilibrium p-value greater than 10^−6^. After SNP quality control, missing genotypes were imputed using MACH[17,18] on acceptable SNPs, Table 1.

**Table 1:** Final participant demographics.

	LABS-PD baseline N = 537 Mean (SD)	Baseline Data for Model N = 244 Mean (SD)	Last Follow-up N = 244 Mean (SD)
**Age (Years)**	59.8 (9.8)	59.60 (8.5)	66.6 (8.5)
**Duration Since Diagnosis (Years)**	0.8 (0.8)	0.8 (0.8)	7.8 (0.8)
**MMSE**	29.3 (1.0)	29.4 (1.1)	28.7 (2.6)
**Total UPDRS**	24.7 (10.1)	22.3 (9.0)	31.3 (16.2)
**UPDRS Motor**	17.4 (8.6)	15.9 (7.0)	21.5 (11.2)
**UPDRS ADL**	6.1 (3.2)	5.8 (3.1)	8.2 (5.4)
**S/E ADL**	93.2 (5.2)	93.1 (5.2)	88.5 (10.6)
**MoCA**	NA	NA	26.6 (3.9)
**GDS**	NA	NA	2.2 (2.4)

MMSE—Mini Mental Status Exam; UPDRS—Unified Parkinson’s Disease Rating Scale; ADL—Activities of Daily Living; S/E ADL—Schwab and England Activities of Daily Living; MoCA—Montreal Cognitive Assessment; GDS-Geriatric Depression Scale

For clinical variable processing, time-varying clinical variables were segmented by time. Next, pruning and imputation of missing data was performed as follows: (1) all variables with more than 5% missing data were removed, (2) all participants with more than 20% missing data were removed, and (3) assuming missingness at random, the remaining missing values were imputed using the R package *imputation* based on *k*-nearest neighbors, where *k* was selected by cross-validation.

The final dataset contained many distinct variable types, some of which required specialized mathematical treatment. The SNP data were divided into two groups: (1) SNPs of known PD interest that were present in the dataset (identified by the investigators and a National Human Genome Research Institute meta-analysis[19]) and (2) all other SNPs. The first group of SNPs was represented explicitly (n = 25) in the model using dominant, recessive, and additive codings, while the second group of SNPs was collapsed into the principal components of genetic data[20]. This method allowed the explanatory power of the genetic components pertaining to the ancestry to be retained, led to explicit analysis of SNPs of known interest, and reduced the number of variables by three orders of magnitude.

Imaging data was acquired on a different schedule from other variables and therefore had to be interpolated around the clinical data collection schedule. This method allowed the dataset to be built around the presence of discrete time steps (annual visits). First, a mixed effects linear model was fit, with participants being the random effect, to the individual imaging variables. Then, the imaging variables were re-sampled at times that matched the visit year using the fitted model.

### Building the statistical model

The REFS modeling process consists of two distinct machine-learning steps–*enumeration* and *optimization*, followed by the process of investigating the model via *simulations*. Enumeration is the process of listing the distinct reasonable small-scale models for each dependent variable in the overall problem. Optimization is the task of subsequently putting it all together in a large optimal network, and REFS provides a number of tools and checkpoints for controlling the quality of the final model such as specific heat to measure model complexity and model score convergence. Once the model is built, it can be investigated via systematic *in silico* simulations.

Fragment enumeration involves the consistent listing and regularized scoring of alternative models describing every dependent variable in the dataset. The goal of this step is to thoroughly explore the universe of ‘building blocks’ for use in optimization, while being subjected to realistic computing run time constraints. The REFS model in this study had 570 variables, so it was explored to a significant depth by allowing up to 4 non-SNP inputs per output. This method resulted in nearly 200 million fragments per output variable being scored and 100 billion for the overall model. After filtering, 5.3 million fragments of all types were advanced to the optimization phase of model building. In that phase, candidate models were evaluated, subject to further regularizing constraints, and an ensemble of 1024 optimized models was eventually constructed by sampling.

### Investigating the model—Simulations

Investigation of REFS models proceeds via a series of in silico simulations, which often encode pairs of perturbation and control experiments. Forward simulations are performed by setting a putative variable to two states, the 5^th^ percentile of the training data, and the 95^th^ percentile. Variables of interest are then read out, and the appropriate statistical analyses are conducted.

Two types of simulations were performed: firstly, focusing on a single baseline predictor, simulations were performed measuring an outcome at a given time (“disease state” simulations); secondly, focusing on a baseline predictor, simulations were performed across a time series of a given outcome variable for years 4–7. These approaches are described in the next two paragraphs.

First, for the disease state simulations, putative predictors were set to 5^th^ and 95^th^ quantiles of training data and the network was allowed to propagate the signal to the endpoints. The so-called Bayesian posteriors (simulated endpoint value distributions) were measured. The statistical significance of the difference between the posteriors of endpoints corresponding to the 5^th^ and 95^th^ quantiles was then assessed by a permutation test, after subsampling the posterior to reduce the total number of points equal to the number of patients in the training data set. P-values were deemed significant below 0.05 and exploratory below 0.1 in accordance with standard statistical practice[21].

Second, all of the values for a given output variable were simulated across the time points, generating rates of progression between follow-up visits. Then, Gaussian linear model fits of variable vs. time were built for every sample of that output variable, and only the distribution of slopes was retained, discarding the intercepts. Monte Carlo sampling was performed to determine the expected perturbation effect significance on the newly computed distribution of slopes. This method of testing was expected to reveal only the strongest and most consistent effects on the rate of disease progression. This statistical test was designed to determine whether a variable drove the rate of disease progression, not whether its initial state was important to the final outcome. Therefore, a perturbation could have a prolonged and significant effect on an endpoint, but fail to show up in results from this line of inquiry. The analysis of the initial state importance was addressed in the disease state simulations.

### Validating the statistical model

Once the basic requirements governing ensemble score have been satisfied, the recall of training data for the output variables of interest is validated. This validation is accomplished by setting all baseline variables and the drug treatment data between the baseline and the time point of interest (since interventions cannot have explainers in the model). Then, the baseline signal is allowed to propagate, and outcome variables of interest are collected (e.g. UPDRS, MoCA, etc.) and compared to the training data value for that variable. Since the signal may propagate through multiple levels of the model, the R^2^ values obtained in this way may differ from those obtained from a simple predictor model: they will generally tend to be slightly lower than those for a comparable linear model involving the baseline variables and the outcome but without any of the intermediate explainers of causality owing to the loss of information in multi-step simulations.

The Michael J. Fox Foundation Parkinson Progression Marker Initiative (PPMI) dataset was used to validate the results[22]. The PPMI is an ongoing cohort study supported by the Michael J Fox Foundation that uses many of the same assessments and outcomes as LABS-PD. Using a cut of the PPMI dataset as obtained on 11/13/2015, we identified 150 unique participants who had been under observation for 4 years or longer (similar to the LABS-PD cohort), who had 3 or more MoCA scores measured in that time, who had APPRDX status flag equal to 1 or 2 (cases or healthy controls), and did not exhibit a change of primary diagnosis from baseline onwards. Additionally, participants were required to have genetic data profiled via Illumina Immunochip. A further cut of the data was assessed on 01/12/2016. Unlike the main model, which was built hypothesis-free within the Bayesian causal modeling framework, the validation effort using the PPMI dataset was performed in a hypothesis-driven manner using a conventional mixed-effects linear model as implemented by the R package *nlme*[23]. We set out to validate the effect of rs11724635, the only SNP implicated in the progression of disease in the causal model. We added primary diagnosis, visit date, and age of onset to the model, allowing for the possible 4-way interaction term and all lower terms, and simplified the model using stepwise search as implemented in *stepAIC* (k = log(n), or BIC-driven) from the package *MASS*[24] in R. Patient ids were used as random effects to allow for patient-specific random intercepts.

**Fig 2:**
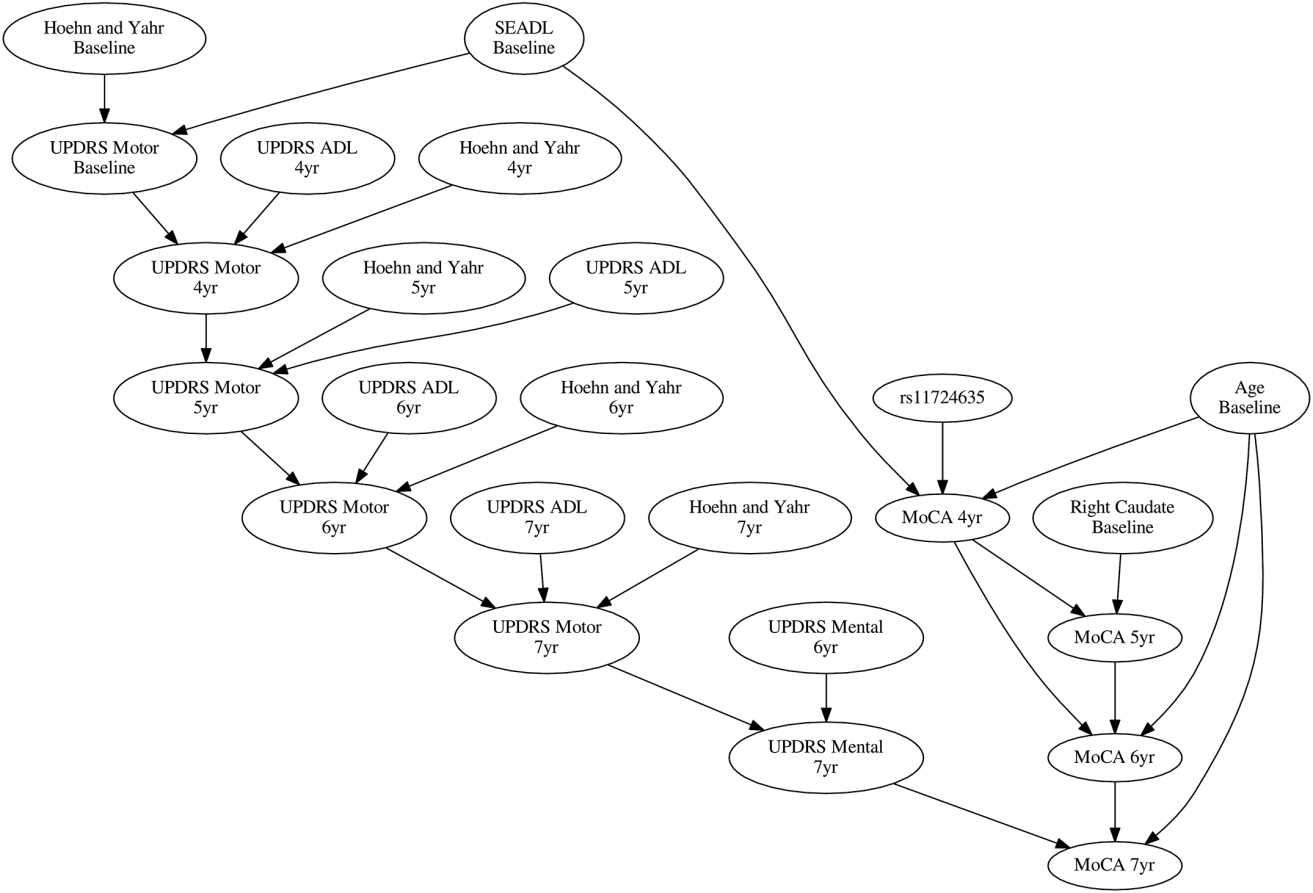
The network diagram of rs11724635. 30% consensus average network of the model ensemble shows the context for rs11724635’s influence on MOCA rate of change. Among baseline variables, caudate imaging was predictive for some MOCA endpoints but not for the overall rate of progression.

Fig 2 offers a broader overview of the causal drivers of MOCA over time and puts the influence of rs11724635 in the context of other variables in the model. Rs11724635 being the only genetic driver of progression in the model (from the few considered), it was chosen for out-of-sample validation using the PPMI data.

## Results

### Disease state simulations

**Fig 3:**
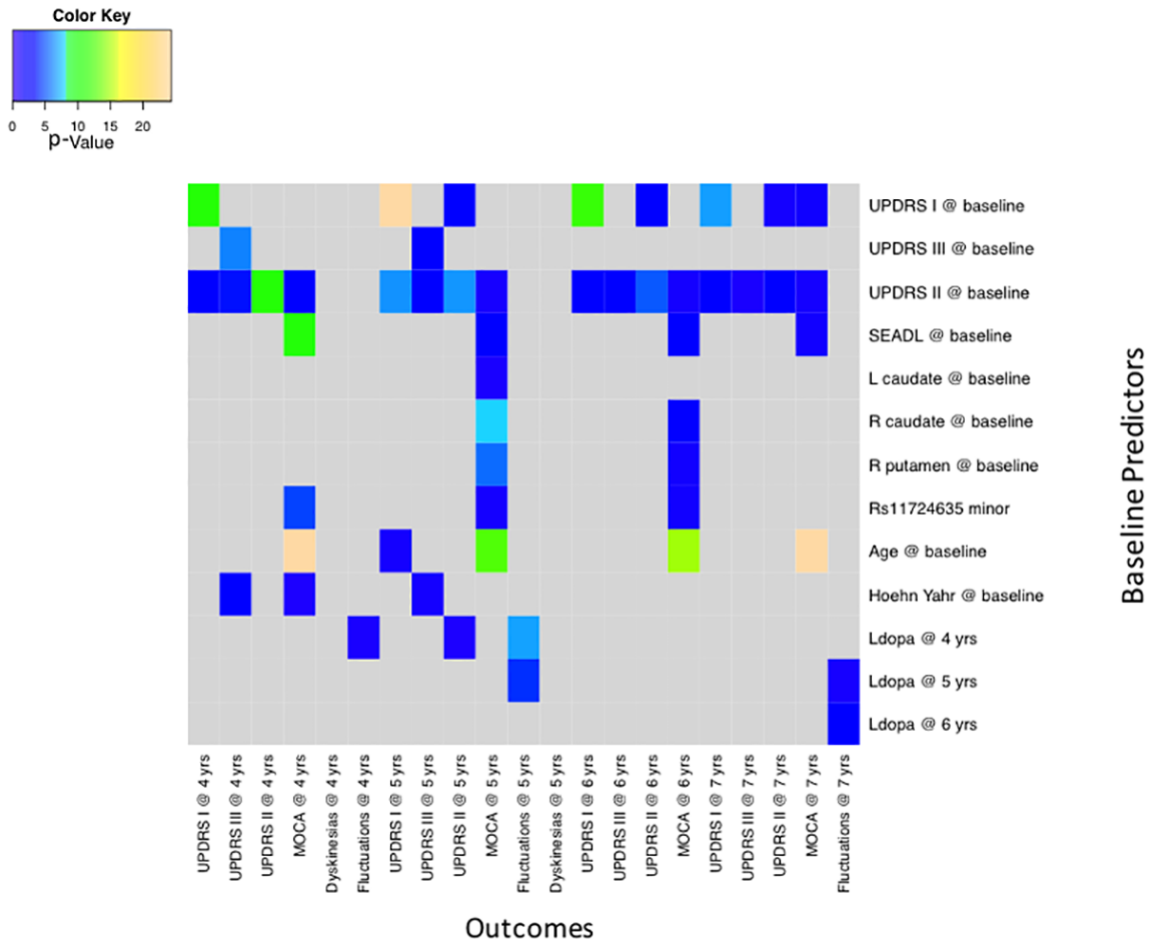
Baseline predictors of study outcomes. log10(p-value) of perturbation effect upon endpoint variables at various time points. The y-axis shows predictors and the x-axis shows the outcomes at different time points. Terrain colors show the level of significance of the relationship between baseline predictors and outcome variables. UPDRS—Unified Parkinson’s Disease Rating Scale: I—Mental, II—Activities of Daily Living, III- Motor Exam; S/E ADL—Schwab and England Activities of Daily Living; MoCA—Montreal Cognitive Assessment; L and R Caudate—DAT scan striatal binding ratios; RS11724635minor—minor allele of the SNP rs11724635; Ldopa—levodopa equivalents.

In disease state simulations, the effect of influencing a predictor variable at baseline was measured on motor and cognitive outcomes at follow-up years 5–7 (Fig 3). Motor performance at years 5, 6 and 7, measured by UPDRS part III, was significantly and consistently associated with baseline ADL, measured by UPDRS part II (p = 0.0007 for year 5, p = 0.0053 for year 6, and p = 0.0736 for year 7). None of the other baseline measures, including baseline UPDRS part III, were consistently predictive of motor outcomes beyond year 5. Motor fluctuations at 5 and 6 years were associated with levodopa equivalents at years 5 and 6.

Two baseline measures, age and S/E ADL score, were significantly associated with cognitive function measured by MoCA at years 5, 6, and 7. Worse MoCA scores were significantly associated with baseline older age (p = 2.7E^-13^ for year 5, 1.44E^-15^ for year 6, and 4.67E^-25^ for year 7) and lower baseline S/E ADL scores (p = 0.0005 for year 5, 0.0019 for year 6, and 0.0389 for year 7). Similar results were seen for UPDRS part II, with p-values reaching the exploratory cut off of <0.1. At years 5 and 6, there were associations of MoCA scores with DAT uptake in the left caudate (p = 0.0693 for year 5 and 0.10 for year 6), the right caudate (p = 1.28E^-8^ for year 5 and 0.0159 for year 6), and the right putamen (p = 2.7E^-6^ for year 5 and 0.0454 for year 6), but this finding was not present in year 7. There was a weak association for the minor allele of the SNP rs11724635, with MoCA scores at years 5, 6 and 7 (p = 2.7E^-5^ for year 5, 0.0533 for year 6, and 0.0424 for year 7). This allele has been associated with risk for for PD[25,26].

### Disease progression simulations

**Fig 4:**
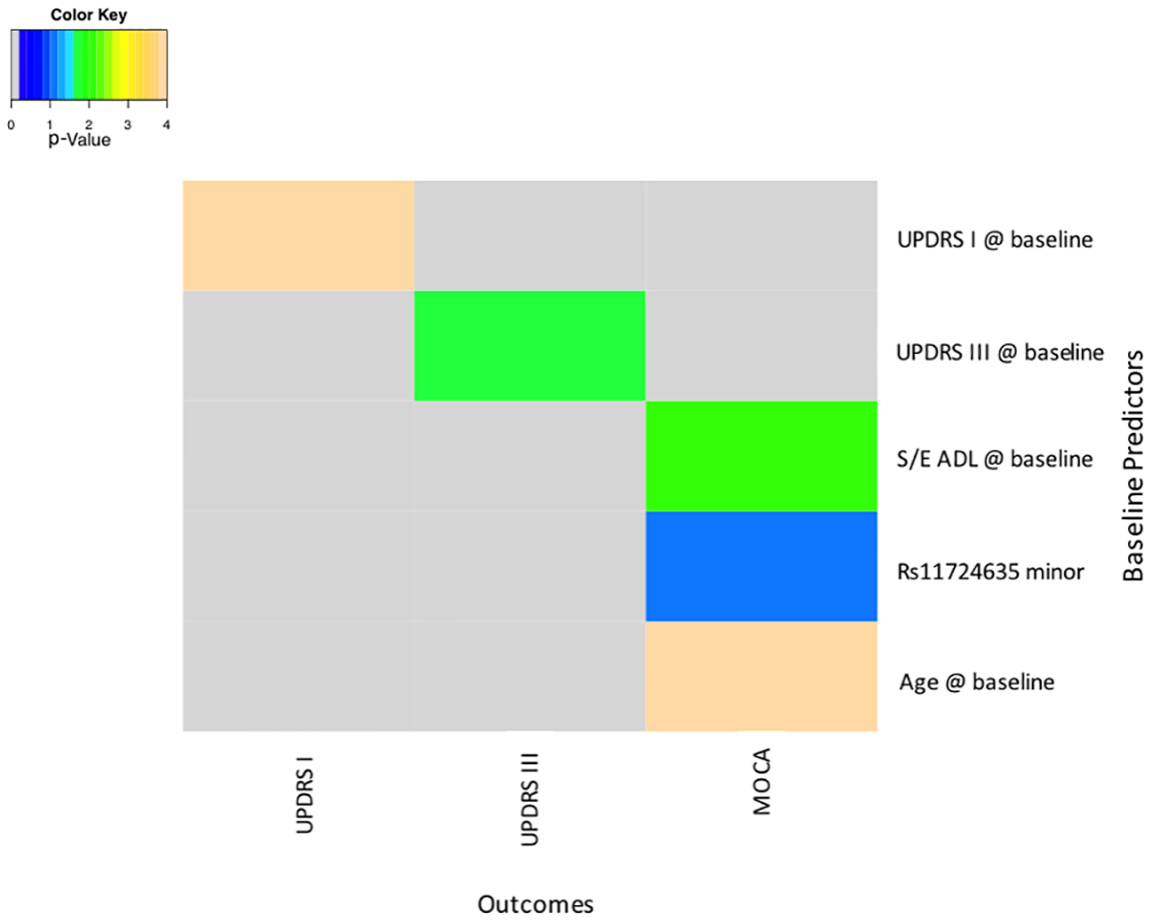
Baseline predictors of rates of change of study outcomes. log10(p-value) of perturbation effect upon endpoint progression. UPDRS—Unified Parkinson’s Disease Rating Scale: I—Mental, II—Activities of Daily Living, III- Motor Exam S/E ADL—Schwab and England Activities of Daily Living; MoCA—Montreal Cognitive Assessment; RS11724635minor—minor allele of the SNP rs11724635.

In disease progression simulations, the effect of perturbing a variable on another variable’s rate of change over time was measured (Figs 3 and 4). The clearest overall progression effect was upon MoCA test results. MoCA rate of progression was influenced by baseline S/E ADL, subject age, and by the SNP rs11724635. Rates of progression of the UPDRS motor and mental subsections, analyzed separately, depended upon baseline measurements of those variables, suggesting an overall acceleration trend in the evolution of symptoms.

### Out-of-sample validation

**Fig 5:**
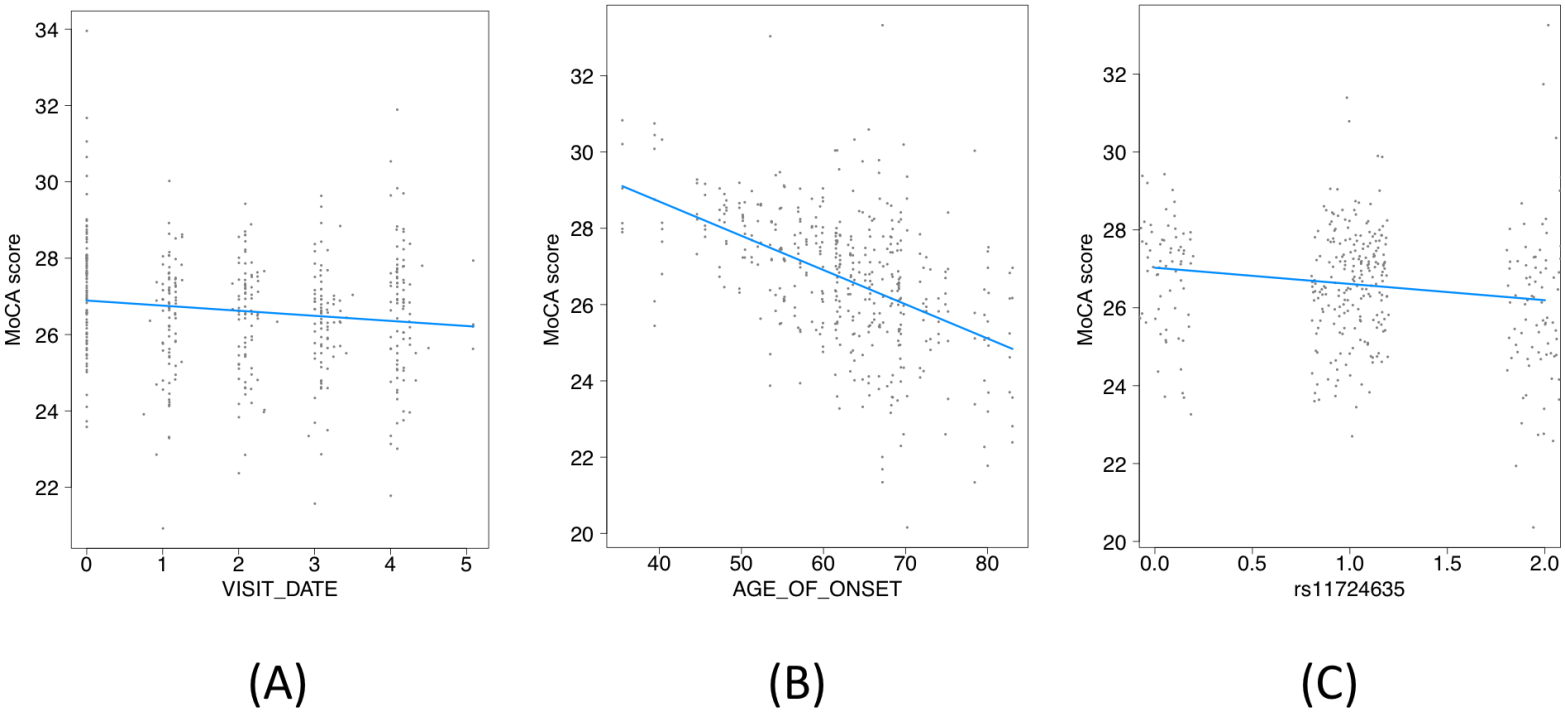
Out-of-sample validation of rs11724635. (A), (B), (C)—Individual contributions to rate of change of MoCA by visit date, age of onset, and rs11724635, including interaction terms (cases only).

Using the PPMI dataset, we set out to validate some key disease progression simulation results: that rate of MoCA progression depended on age of onset and on the level of rs11724635 (Fig 5, Table 2). Although we knew the predicted directions of correlation, non-directional p-values were reported in Table 2. Thus, the significances of validation terms, e.g. **Primary Diagnosis: Visit Date**:rs11724635, are reported as ~2x as high as the expected directional p-values. Using this dataset, we were able to validate both hypotheses at the significance level of 0.05. Furthermore, as predicted, age of onset was a far stronger predictor of the rate of decline of MoCA scores than rs11724635. Nevertheless, the genetic signal is of considerable interest as a potential prognostic marker for cognition.

**Table 2:** Details of the validation model, cases and controls, stratified by primary diagnosis.

	numDF	denDF	F-value	p-value
**(Intercept)**	1	593.000	34821.997	0.000
**Primary Diagnosis**	1	144.000	10.253	0.002
**Primary Diagnosis: Visit Date**	2	593.000	3.143	0.044
**Primary Diagnosis: Age of Onset**	2	144.000	11.940	0.000
**Primary Diagnosis:rs11724635**	2	144.000	1.179	0.311
**Primary Diagnosis:Visit Date:Age of Onset**	2	593.000	4.059	0.018
**Primary Diagnosis:Visit Date:rs11724635**	2	593.000	2.462	0.086

ANOVA table for the simplified validation linear model. Slopes are not shown due to aggregation across the discrete levels of diagnosis, in favor of showing overall p-values. Note that these p-values reflect two-tailed comparisons to null whereas the slopes were known from training data and therefore the p-values may be considered to be roughly 2x higher than their actual values. The 3-way interaction term with the diagnosis, visit, and SNP is significant at < 0.1 without taking this into account and at < 0.05 if this adjustment is made. In other words, there may be a disease-specific MoCA decline with observation time that is further modified by the level of rs11724635

It is worth repeating here that the SNP selection strategy for the hypothesis-free causal model was not, in itself, hypothesis free—we only retained SNPs previously implicated in Parkinson’s disease onset in the literature subject to QC criteria. A broader comparison of genetic markers between the LABS-PD and PPMI studies is warranted and, indeed, planned by the authors.

## Discussion

We found several baseline predictors of motor and cognitive outcomes at 5 or more years of follow-up. In particular, functional status as measured by activities of daily living at baseline (UPDRS ADL section and the S/E ADL scale) were consistent and relatively strong predictors of motor and cognitive performance at 5, 6 and 7 years. Older age at baseline was predictive of worse cognitive outcome. Lower DAT uptake at baseline, particularly in the caudate nuclei, and the presence of the minor allele of rs11724635 may also contribute to worse long-term cognitive outcomes.

These findings show that machine-learning Bayesian network methods that incorporate imaging, DNA, or biological samples can be a powerful complement to classical statistical analyses for identifying factors affecting outcomes and progression of PD. The replication of established associations such as age and cognitive function, and levodopa equivalents and motor fluctuations, supports the validity of this modeling approach. The relationship between advanced age and worse cognitive outcomes has been shown in several cohorts[27]. Additionally, levodopa dose, expressed here as levodopa equivalents, is an established risk factor for motor complications. Causal models strive to separate upstream (‘causal’) predictors from downstream (‘correlated’) predictors, and they tend to have lower false-positive rates with respect to mechanistically relevant targets. Since the REFS platform is a causal model, we expect findings to be enriched for disease-relevant correlations. The simulation results confirm prior findings generated from more traditional statistical methods and point to new relationships and potential pathways for understanding progression.

UPDRS part II and S/E ADL, composite measures that incorporate motor function and other cognitive and behavioral factors, were consistent predictors of motor and cognitive outcomes. Additionally, these two measures monitor function over two weeks, as compared to a one-time motor or cognitive exam. Thus, these may be more reliable assessments for predicting motor function than a single examination. The predictive value of these functional tests for cognitive and behavioral outcomes may be explained in a similar way. Baseline MMSE was included among potential predictors, but was not significant. Baseline MMSE may be insensitive to early cognitive deficits in PD[28], particularly the executive dysfunction common in this disorder, while modest reductions in executive function may be reflected in ADLs. Similarly, even mild depression in PD is independently associated with worse self-report motor function and ADLs[29]. Baseline ADLs have independently been associated with a broad range of key clinical and practical milestones, such as the initiation of symptomatic therapy in the PPMI cohort (Personal communication, Tanya Simuni) and cessation of driving[30]. These findings suggest that synthetic measures like UPDRS part II and S/E may be better prognostic variables than measures of individual PD features or domains. For long-term trials, such as the NINDS supported NET-PD LS-1 trial[31], it may therefore be useful to adjust for baseline self-reported function, even when starting with de novo subjects.

Age was strongly associated with cognitive function, but not predictive of motor outcomes. Several studies have addressed the role of age in PD severity with mixed results. Clearly age is the single strongest risk factor for PD, and it is related to motor severity at baseline[27]. However, our results suggest that age may not predict future motor outcomes. These results do show that cognitive outcomes are worse with advanced age and this is consistent with multiple studies such as the Campaign study[32,33]. There may be several reasons for this divergence of motor and cognitive associations with age. Dopaminergic therapies may mask the relationship with motor function as compared to generally untreated cognitive decline. Additionally age-related processes may cause cognitive decline such as concomitant Alzheimer’s disease or vascular pathology[34].

The association of baseline DAT imaging with cognitive outcomes was previously shown in this cohort using more traditional statistical models[35]. In the prior analyses both caudate and putamen DAT binding were associated with MMSE and MoCA scores up to approximately 5 years of follow-up. The current analyses confirm this finding using an unbiased approach and showed an association at 6 years for the left caudate only. Prior associations with baseline DAT uptake and motor function were not replicated in this Bayesian model and may be accounted for by the associations with baseline functional status which was not found in prior hypothesis driven analyses.

Among the genetic markers of risk included in the analysis the only relationship found was with the SNP rs11724635; the minor allele was associated with faster cognitive progression. The SNP rs11724635 is located in the gene bone marrow stromal cell antigen 1 (BST1) gene, and is named for its role in B cell maturation. This is intriguing given that recent evidence suggests that much of the downstream neurodegeneration in PD may result from inflammatory responses and an interaction of alpha-synuclein and the adaptive immune system[36]. BST1 is also involved in calcium signaling in the endoplasmic reticulum. Polymorphisms in the BST1 gene have been inconsistently associated with risk for PD[37], but a large meta-analyses indicated that the minor allele is associated with a reduced risk for PD[16,19]. A recent small study suggested a possible interaction of the minor allele with well water implicating a role in modulating the effects of environmental toxins[26]. Given the inconsistent population genetics for risk and the small sample size of this study, these findings should be considered exploratory even though they were replicated in a sample of the PPMI cohort. Notably we did not find associations of cognitive decline with ApoE isoforms or variants in MAPT or SNCA, which have previously been reported[32,38,39]. There are several potential reasons for the differences with other studies, including use of a clinical trial population, different stages of disease, assessed SNPs and different cognitive measures, and the unbiased analytical approach.

There are several important limitations of this study. We used a clinical trial followed by an observational cohort wherein assembly of the data frame and need for complete data required further reduction of the sample size. This may have introduced bias by removing subjects if incomplete data was associated with different prognoses than for the subjects who were retained. The direction of bias is difficult to predict, but may reduce the ability to detect associations by reducing heterogeneity in the spectrum of clinical measurements. However, the clear replication of established associations shows the validity of this model approach. This approach may be of greatest use in large multiplex datasets where multiple biomarkers or clinical factors of interest are prohibitive for traditional hypothesis driven analyses. The MJFF funded PPMI effort is a clear example of this kind of dataset. As the first step, we used an early slice of the PPMI dataset to validate some of the findings of the current study. PPMI and future longitudinal OMICS studies may benefit from the analytical approach described in this paper.
